# Ononin Inhibits Tumor Bone Metastasis and Osteoclastogenesis By Targeting Mitogen-Activated Protein Kinase Pathway in Breast Cancer

**DOI:** 10.34133/research.0553

**Published:** 2024-12-16

**Authors:** Kumar Ganesan, Cong Xu, Song Wu, Yue Sui, Bing Du, Jinhui Zhang, Fei Gao, Jianping Chen, Hailin Tang

**Affiliations:** ^1^ School of Chinese Medicine, LKS Faculty of Medicine, The University of Hong Kong, Hong Kong, China.; ^2^ State Key Laboratory of Oncology in South China, Guangdong Provincial Clinical Research Center for Cancer, Sun Yat-Sen University Cancer Center, Guangzhou, Guangdong, China.; ^3^ College of Food Science, South China Agricultural University, Guangzhou, Guangdong, China.; ^4^ Laboratory of Southwestern Chinese Medicine Resources, School of Pharmacy, Chengdu University of Traditional Chinese Medicine, Chengdu, China.

## Abstract

Breast cancer (BC) often spreads to bones, leading to bone metastasis (BM). Current targeted therapies have limited effectiveness in the treatment of this condition. Osteoclasts, which contribute to bone destruction, are crucial in supporting tumor cell growth in the bones. Breast cancer bone metastasis (BCBM) treatments have limited efficacy and can cause adverse effects. Ononin exhibits anticancer properties against various cancers. The study examined the impact of ononin on the BCBM and the signaling pathways involved. Our study utilized a variety of experimental techniques, including cell viability assays, colony formation assays, wound-healing assays, Transwell migration assays, Western blot analysis, and tartrate-resistant acid phosphatase (TRAP) staining. We examined the effects of ononin on osteoclastogenesis induced in MDA-MB-231 conditioned medium- and RANKL-treated RAW 264.7 cells. In a mouse model of BCBM, ononin reduced tumor-induced bone destruction. Ononin treatment effectively inhibited proliferation and colony formation and reduced the metastatic capabilities of MDA-MB-231 cells by suppressing cell adhesion, invasiveness, and motility and reversing epithelial–mesenchymal transition (EMT) markers. Ononin markedly suppressed osteoclast formation and osteolysis-associated factors in MDA-MB-231 cells, as well as blocked the activation of the mitogen-activated protein kinase (MAPK) pathway in RAW 264.7 cells. Ononin treatment down-regulated the phosphorylation of MAPK signaling pathways, as confirmed using MAPK agonists or inhibitors. Ononin treatment had no adverse effects on the organ function. Our findings suggest that ononin has therapeutic potential as a BCBM treatment by targeting the MAPK pathway.

## Introduction

Breast cancer (BC) is among the most commonly diagnosed malignancies globally, with an incidence rate of 11.5%, ranking second only to lung cancer [1]. BC has the potential to metastasize to various organs, and bone metastasis (BM) accounts for approximately 50% of cases [2]. BM often leads to tumor-associated bone pathologies, markedly reducing patients’ quality of life. Patients with BC and BM have a median survival period ranging from 24 to 36 months [3,4]. Current treatments for BCBM have limited effectiveness and focus primarily on palliative care [5]. However, these treatments are associated with various side effects, including renal toxicity and jaw osteonecrosis [5]. Currently, there is no ideal solution for preventing BC from metastasizing to bones. The prevailing treatment strategy entails the excision of BMs through surgery and augmentation with chemotherapy. However, this method presents noteworthy perioperative risks and often yields suboptimal results.

The onset of BCBM is a multifaceted process that involves bone cells and cancer cells, resulting in the disruption of normal bone remodeling mechanisms. Hence, it is crucial to concurrently suppress metastasis and bone deterioration during treatment [6]. Bisphosphonates have shown positive effects in BM management [3], and denosumab was developed to curb bone loss and skeletal-related events [7]. However, a recent phase 3 clinical study reported that denosumab treatment did not markedly improve survival rates in the management of BCBM [8]. Currently, most patients with BC and BM receive palliative treatment [9], making it urgent to identify new drugs or strategies against BCBM. Comprehending the mechanisms and factors driving BCBM is crucial for devising effective prevention and treatment strategies.

Tumor-driven osteoclastogenesis is critical in BCBM [4]. In BC, tumor cells engage with bone cells to stimulate the activation of osteoclasts [5]. This interaction results in excessive bone degradation and the secretion of growth factors (GFs), which subsequently fosters tumor proliferation and metastasis [10]. Osteolytic lesions are predominantly observed in the majority of patients with BC, whereas osteoblastic lesions occur in only 15 to 20% of patients [11]. The formation of metastatic bone lesions is a complex process involving various chemokines and cytokines [12,13], leading to continuous tumor progression and bone destruction. Interactions between tumor cells and osteoblasts or osteoclast precursors play a pivotal role in BCBM [14]. Therefore, inhibition of osteoclastogenesis is a promising approach to prevent or treat BM in patients with BC.

Furthermore, several signaling pathways, such as osteoprotegerin (OPG), receptor activator of nuclear factor κB (RANK), and receptor activator of nuclear factor κB ligand (RANKL), TGF-β, mitogen-activated protein kinase (MAPK), and Wnt signaling pathways, are important in BCBM [15] and have been verified to play a crucial role in the BCBM process. Various chemokines [chemokine (C–C motif) ligand 2 (CCL2), CCL3, interleukin-1/6/8/11 (IL-1/6/8/11), and chemokine (C–X–C motif) ligand 12 (CCL12)] [16] and cytokines [RANKL, parathyroid hormone-related protein (PTHrP), prostaglandin E_2_ (PGE_2_), tumor necrosis factor (TNF), and macrophage colony-stimulating factor (M-CSF)] [7,17,18] have been reported to be key ligands in the signaling pathway. RANK and CXCR4 were the predominant receptors. The MAPK pathway is a crucial signaling cascade that mediates several cellular activities, such as proliferation, differentiation, and survival.

Ononin is a specific flavonoid compound primarily observed in the root nodules of legume plants [4]. These include clover (Trifolium spp.), *Astragalus membranaceus*, *Sophora flavescens*, *Ononis spinose*, *Ononis angustissima*, *Smilax scobinicaulis*, *Millettia nitida*, and soybeans (*Glycine max*) [19]. In addition to legumes, ononin is found in various vegetables and fruits. Studies have shown that it possesses antitumor, anti-inflammatory, and protective properties [20–25]. Moreover, studies have demonstrated that it can potentially inhibit tumor metastasis [26,27]. Earlier, our study found that ononin has the potential to prevent triple-negative breast cancer (TNBC) lung metastasis and the associated molecular pathways [28]. Our present findings suggested that ononin exerts noteworthy inhibitory effects on the migration, invasion, and colony formation of human BC cells in vitro. Additionally, it effectively suppressed tumor-induced osteoclastogenesis and inhibited RANKL-induced differentiation of osteoclasts from RAW 264.7 cells in vitro. Moreover, administration of ononin markedly ameliorated tumor-induced osteolysis. These findings demonstrate that ononin can markedly inhibit osteoclastogenesis and osteolysis associated with metastatic BC in vivo and curtail the migration, invasion, and colony formation of BC cells. Given these robust results, ononin has emerged as a promising therapeutic candidate for the treatment of BC with BM.

## Results

### Ononin inhibits the malignant phenotypes of TNBC cells

**Fig. 1.**
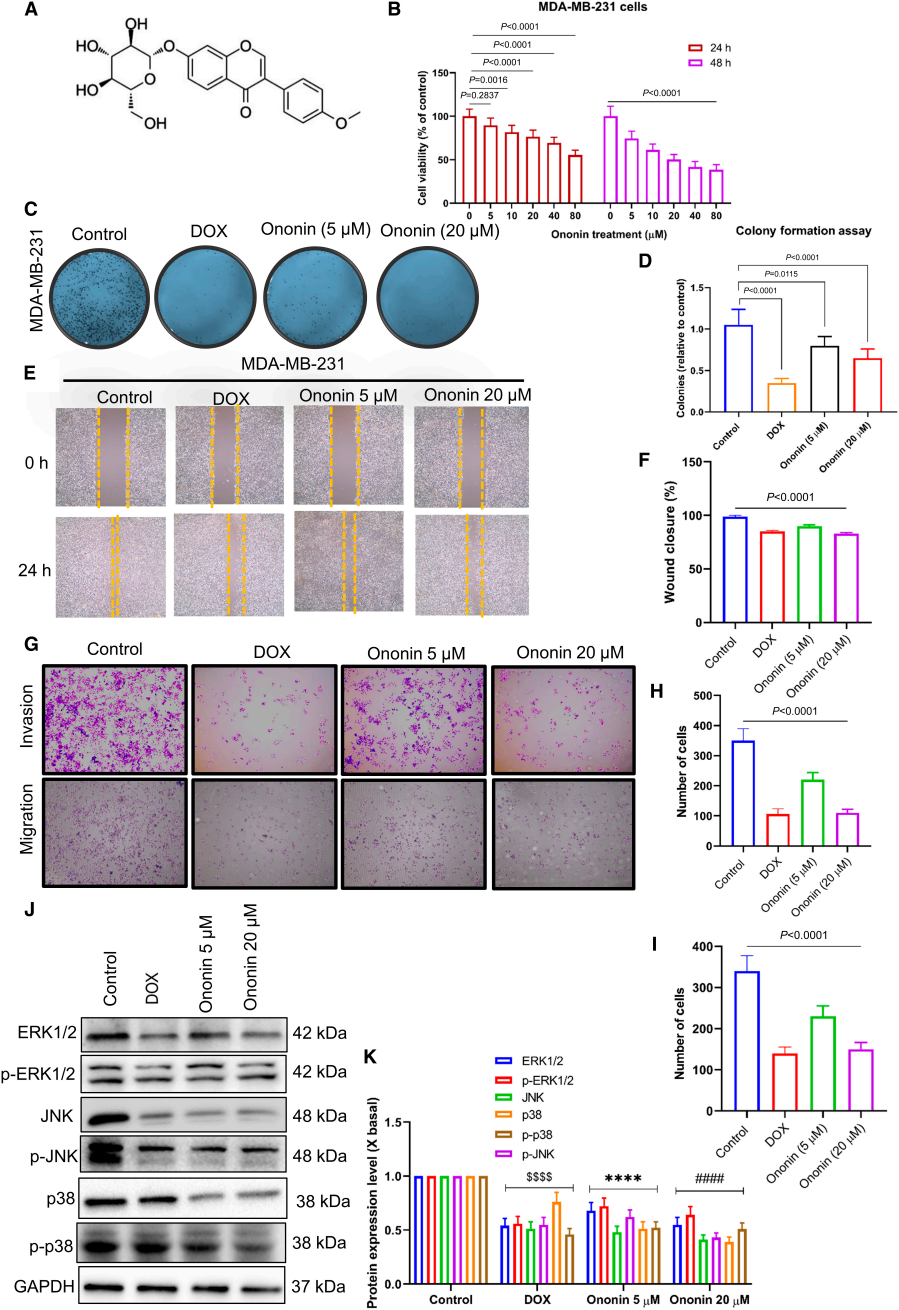
Antiproliferative effects of ononin on MDA-MB-231 cell lines. (A) Chemical structure formula of ononin. (B) Ononin was administered to MDA-MB-231 cell lines at concentrations ranging from 0 to 80 μM over periods of 24 and 48 h, subsequent to which MTT assays were conducted. (C) Results from the clonogenic assays were recorded for these cells after exposure to various concentrations of ononin and DOX. (D) Colonies were quantified after exposure to ononin and DOX at the specified concentrations. (E) Wound-healing assay results following separate treatments of cells with DOX and ononin (5/20 μM). (F) The wound closure percentage was calculated. (G to I) Transwell assay results following separate treatments of cells with DOX and ononin (5/20 μM). (J and K) WB results of ERK1/2/JNK/p38 signaling pathway following separate treatments of cells with DOX and ononin (5/20 μM). ^$$$$^ *P* < 0.0001, ****P < 0.0001, ^####^ *P* < 0.0001.

Figure 1A shows the chemical structure of ononin. The 3-(4,5-dimethylthiazol-2-yl)-2,5-diphenyltetrazolium bromide (MTT) assay results indicate that, with the exception of the 5 μM concentration of ononin, which did not markedly affect cell viability after 24 h of treatment, the viability of TNBC cells markedly decreased following 24 or 48 h of treatment with ononin at concentrations ranging from 10 to 80 μM (Fig. 1B). Following 24 h of treatment, the IC_50_ (median inhibitory concentration) values ranged between 40 and 60 μM. In comparison to MDA-MB-231 cells, ononin exhibited markedly lower cytotoxic effects on normal mammary epithelial cells after the same duration of treatment (Fig. S1). Figure 1C and D depict the findings from clonogenic assays carried out on MDA-MB-231 cells. Ononin (at doses of 5 and 20 μM) and doxorubicin (DOX) led to a progressive decrease in colony formation. Figure 1E and F demonstrates that both DOX and ononin treatments effectively inhibit cell migration. The Transwell assay results indicate that ononin treatment can inhibit both cell migration and invasion, with the inhibitory effect being more pronounced at a concentration of 20 μM (Fig. 1G and H). To further investigate the anticancer mechanism of ononin, we detect the protein expression of the MAPK pathway in MDA-MB-231 cells. The results demonstrated that ononin treatment markedly down-regulated the expression levels of p38, c-Jun N-terminal kinase (JNK), extracellular signal-regulated kinase 1/2 (ERK1/2), and their phosphorylated forms (Fig. 1J and K).

### Ononin reverses EMT process and effectively inhibits MMP-2/9 in MDA-MB-231 cells

**Fig. 2.**
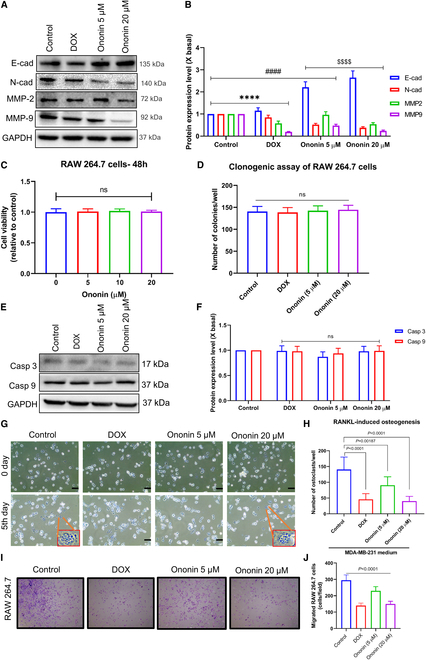
Ability of ononin to suppress EMT evaluated by treating the cells with ononin and DOX. (A and B) WB analysis was employed to assess the expression of E-cad, N-cad, MMP-2, and MMP-9 in TNBC cells. ****P < 0.0001, ^####^ *P* < 0.0001, ^$$$$^ *P* < 0.0001. (C) MTT assays demonstrated that ononin (0 to 20 μM) does not cause cytotoxicity in RAW 264.7 cells after 48-h treatment. (D) Clonogenic assay of control and experimental groups of RAW 264.7 cells. (E) Ononin does not alter caspase-3 and caspase-9 in control and experimental groups of RAW 264.7 cells. (F) Quantification of the apoptosis markers. (G and H) Ononin inhibits RANKL-induced osteoclastogenesis in RAW 264.7 cells (*n* = 6). (I and J) The antimigratory properties of ononin were assessed using a Matrigel-coated Transwell assay on RAW 264.7 cells treated with CM.

Using Western blot (WB) analysis, we assessed the impact of ononin on epithelial–mesenchymal transition (EMT) in TNBC cells by examining cadherins and matrix metalloproteinases (MMPs). The results indicated that ononin treatment led to an increase in the epithelial marker E-cadherin expression, while the mesenchymal marker N-cadherin showed a decrease, suggesting a reduction in mesenchymal characteristics. Concurrently, there was a noteworthy down-regulation of MMP-2 and MMP-9, with MMP-9 being particularly affected. This indicates important modulation of biomarker expression, which is consistent with the reduction in mesenchymal traits (Fig. 2A and B). These findings suggest that ononin’s capacity to facilitate a shift from a mesenchymal to an epithelial state could underlie its effectiveness in reducing the metastatic propensity of TNBC cells.

### Ononin demonstrates nontoxicity in RAW 264.7 cells

RANKL-induced RAW 264.7 cells have been established as a valuable tool for investigating bone homeostasis [29–31]. In this study, we investigated whether ononin exhibits any toxic side effects on RAW 264.7 cells. Figure 2C presents the outcomes of MTT assays. After a 48-hour treatment with ononin at concentrations of 5, 10, and 20 μM, cell viability remained unimpaired. Figure 2D shows that no notable dose-dependent variations in colony formation were detected. Further analysis via WB to detect caspase-3 and caspase-9 protein expression revealed that ononin had no impact on the expression of these apoptosis markers in RAW 264.7 cells (Fig. 2E and F). These findings strongly indicated that ononin does not induce any toxic effects in RAW 264.7 cell lines.

### Ononin inhibits osteoclastogenesis in RAW 264.7 cells induced by tumor cells through ERK1/2/JNK/p38 pathways

**Fig. 3.**
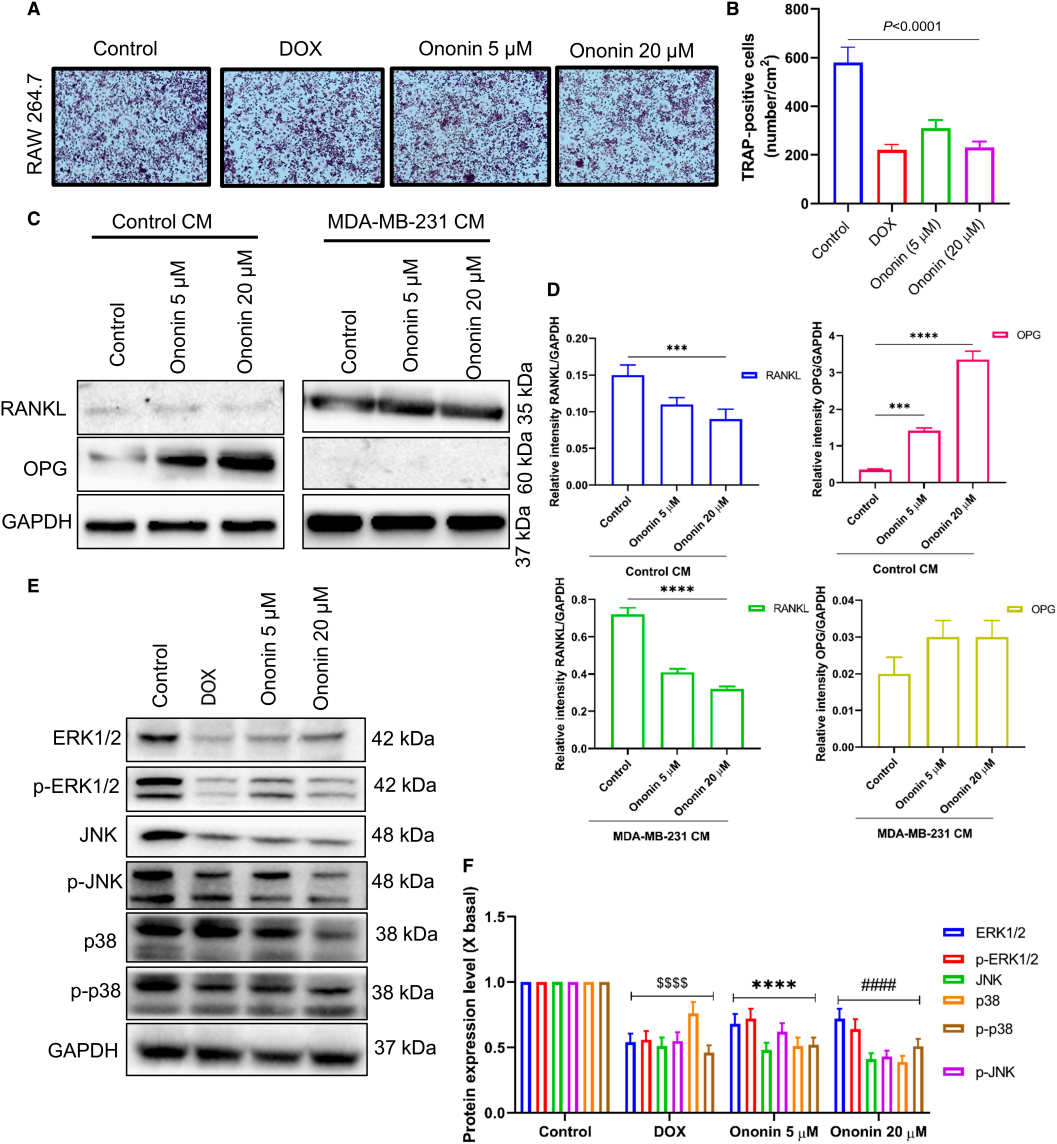
(A and B) Ononin suppresses osteoclast differentiation induced by CM, as demonstrated by TRAP staining assay. (C) Effects of ononin on control CM [Dulbecco’s modified Eagle’s medium (DMEM)] and MDA-MB-231-induced RANKL and OPG protein expression levels. (D) The relative ratio of RANKL/glyceraldehyde-3-phosphate dehydrogenase (GAPDH) and OPG/GAPDH protein expression was calculated with a densitometer. (E) The assay of JNK/ERK/p38 pathways was performed using a WB. (F) Quantification of the ERK/JNK signaling pathways. ^$$$$^ *P* < 0.0001, ****P < 0.0001, and ^####^ *P* < 0.0001.

To investigate the effects of ononin on the interactions between tumor cells and bone cells, we assessed its impact on osteoclastogenesis and migratory capacity of RAW 264.7 cells activated by conditioned medium (CM) from MDA-MB-231 cells. After stimulating RANKL-treated RAW 264.7 cells with CM, a decrease in osteoclast count per well was noted following pretreatment with ononin (5 and 20 μM) or DOX. This decrease was dose dependent, as shown in Fig. 2G and H. Figure 2I and J demonstrates that the administration of ononin or DOX considerably decreased the migration capabilities of RAW 264.7 cells. Then, we conducted a quantitative analysis of osteoclast differentiation using TRAP staining. RAW 264.7 cells were treated with RANKL and stimulated with CM, followed by pretreatment with ononin (5 and 20 μM) or DOX. Subsequently, TRAP-positive cells were counted under a microscope. The results indicated that ononin inhibits osteoclastogenesis in RAW 264.7 cells induced by CM (Fig. 3A and B). Furthermore, Fig. 3C and D illustrates that cotreatment with ononin (5 and 20 μM) alongside MDA-MB-231 CM markedly suppresses factors associated with osteolysis, particularly RANKL, and enhances bone regeneration, indicated by elevated OPG levels compared with control CM.

Focusing on the MAPK signaling pathways may represent a promising therapeutic approach to enhance bone synthesis while inhibiting osteoclastogenesis and bone degradation in patients with BM [32]. Ononin demonstrates potential as a therapeutic candidate for bone-related disorders, including BM, by enhancing bone regeneration. Treatment with ononin at 5 and 20 μM, along with DOX, markedly down-regulated the expression of ERK1/2, JNK, p38, and their phosphorylated form in in RAW 264.7 cells treated by CM (Fig. 3E and F). These findings propose that ononin beneficially impacts bone integrity by restraining osteoclastogenesis and stimulating bone development through ERK1/2/JNK/p38 pathways.

### Ononin alleviates tumor-induced osteolytic degradation and inhibits tumor metastasis in vivo

**Fig. 4.**
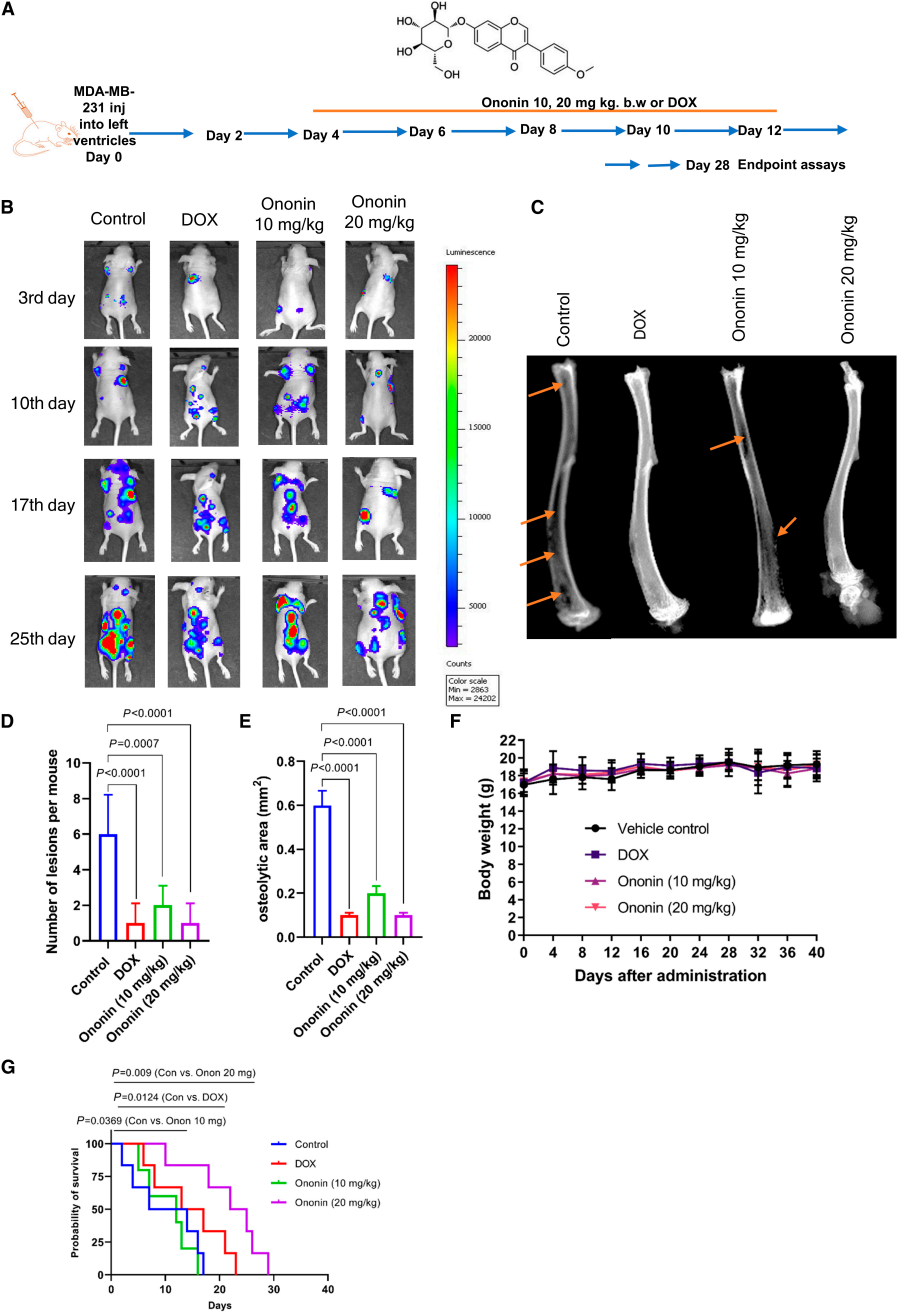
(A) Diagram of the administration of ononin or DOX in the BCBM mouse model. (B) Bioluminescence imaging was conducted on days 3, 10, 17, and 25 following the implantation of cancer cells. (C) Representative radiograph images from mice administered with ononin or DOX. Arrows on the image mark the osteolytic bone lesions resulting from the injection of MDA-MB-231 cells. (D) The lesion count per mouse was determined through analysis of radiographic images from mice administered with ononin or DOX. (E) Osteolytic area (mm^3^) in mice treated with ononin or DOX. (F) Animals’ body weight throughout the study period of up to 40 d. (G) The survival curve was plotted based on the survival of the animal exhibiting BMs after treatment with ononin and DOX.

**Fig. 5.**
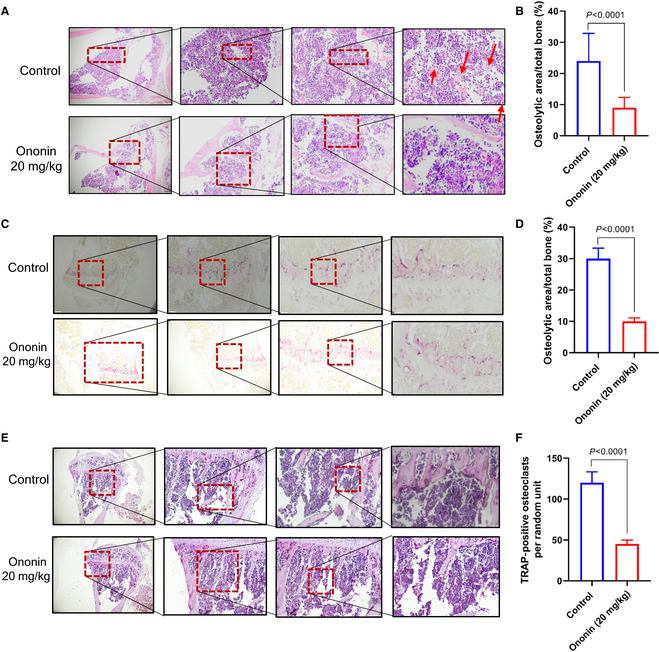
(A and B) H&E staining was employed to assess the impact of ononin treatment on bone tissue. (C to F) Results from TRAP staining indicated a substantial reduction in metastatic tumor load in groups treated with ononin.

Given the observed compound’s suppressive impact on tumor malignant phenotypes and its inhibition of osteoclastogenesis in vitro, we established an animal model using left ventricle injection (1 × 10^6^ per mouse). The treatment protocol is illustrated in Fig. 4A. Using bioluminescence imaging, the inhibitory effects of ononin and DOX on metastasis in BC were observed in mice implanted with MDA-MB-231-luc (*n* = 6) (Fig. 4B). X-ray imaging of leg bones demonstrated that ononin (10 and 20 mg/kg body weight) or DOX markedly decreased the incidence and size of osteolytic lesions (Fig. 4C to E). The various treatments did not have a noteworthy impact on the body weight of the mice (Fig. 4F). Results from hematoxylin and eosin (H&E) staining indicated that the ononin-treated mice exhibited markedly less bone destruction and BM (Fig. 5A and B). Consistent results were observed with TRAP staining, where the ononin-treated mice displayed fewer osteolytic areas and TRAP-positive cells, indicating an inhibition of tumor metastasis (Fig. 5C to F). Moreover, the survival rates of MDA-MB-231 BC-bearing mice were enhanced following treatment with ononin or DOX, as indicated by the survival curves (Fig. 4G). The experimental outcomes demonstrate that ononin effectively alleviates tumor-induced osteolytic degradation and inhibits tumor metastasis in systemic mouse models of metastasis.

### CC-401 is synergistically active with ononin to reduce BCBM

Previously, we investigated the effects of ononin on the MAPK pathway. To substantiate these results, we explored the impact of combining ononin and JNK inhibitor (JNKi) CC-401, a selective adenosine triphosphate (ATP)-competitive anthrapyrazolone MAPK inhibitor, on both the metastatic capability and signaling pathways involved. CC-401 is a potent inhibitor of all 3 JNK isoforms and other kinases, which exhibited strong inhibitory effects. Moreover, it selectively targets JNK and related kinases with at least 40-fold selectivity. High JNK expression was independently associated with unfavorable survival outcomes. Therefore, blocking the JNK signaling pathway at an upstream stage effectively impedes the advancement of experimental mammary tumors toward metastasis.

**Fig. 6.**
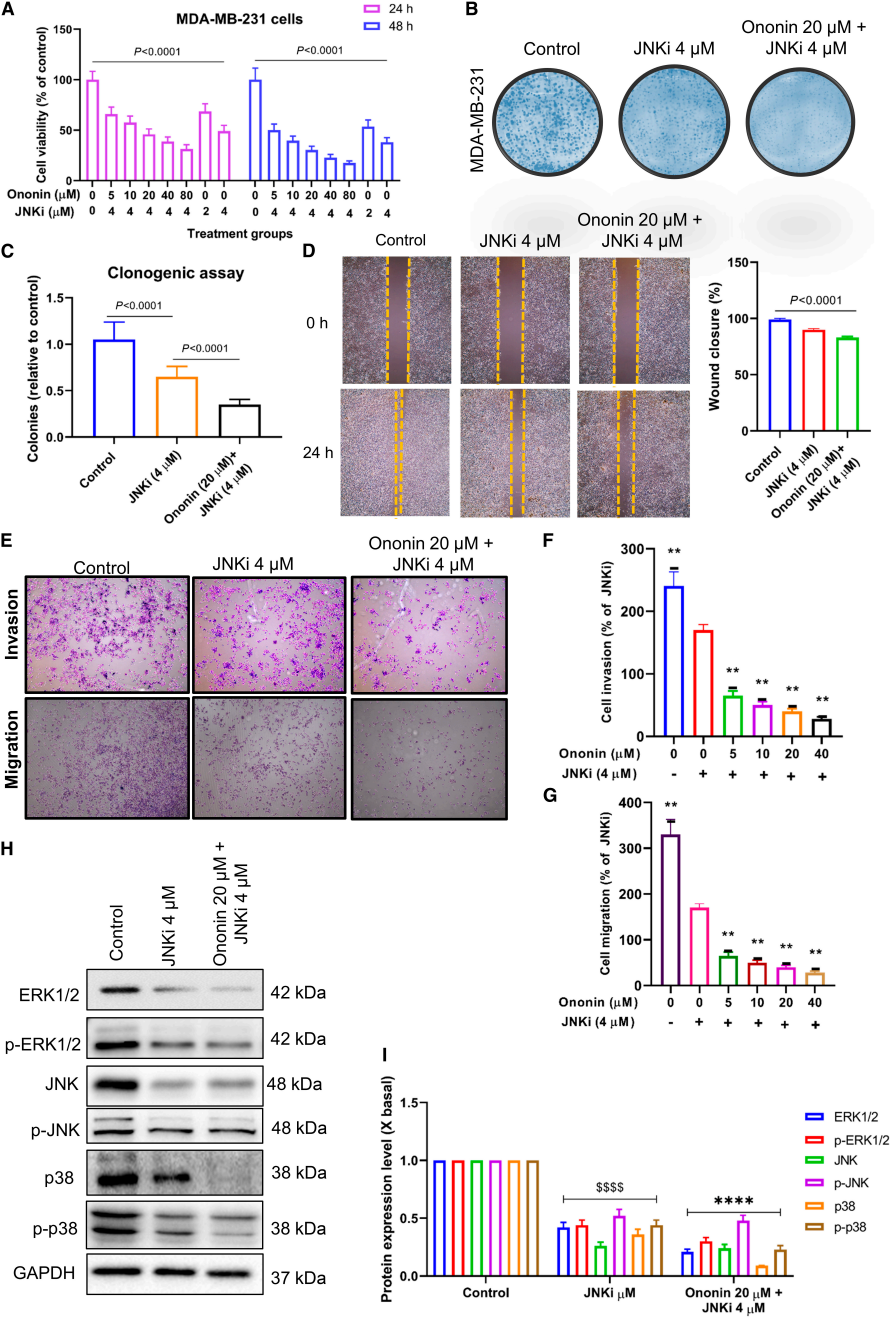
CC-401 is synergistically active with ononin to exhibit inhibitory effects on TNBC cells. (A) Results of cell viability analysis following treatment with various concentrations of ononin in combination with JNKi for 24 and 48 h. (B) Results of the colony formation assay following treatment of cells with JNKi alone and in combination with ononin. (C) Number of colonies was quantified. (D) Results of the wound-healing assay following cell treatment with JNKi alone and in combination with ononin. (E to G) Results of the Transwell assay following cell treatment with JNKi alone and in combination with ononin. *P* < 0.0001, ****P < 0.0001. (H) The assay of JNK/ERK/p38 pathways was performed using a Western blot. (I) Quantification of the ERK/JNK signaling pathways. *P* < 0.0001, ^****^ *P* < 0.0001.

The MDA-MB-231 cells were concurrently treated with ononin and 4 μM CC-401. The dose of JNKi was selected due to its established specificity and documented impacts on BC cells [33]. Our findings indicated that combined treatment with ononin and JNKi markedly enhances the cytotoxic effects on MDA-MB-231 cells compared to the treatment with JNKi alone (Fig. 6A). Furthermore, administering ononin (20 μM) together with JNKi (4 μM) suppressed colony forming when compared to treatments involving only JNKi or the untreated control group (Fig. 6B and C). Figure 6D to G indicates that combined treatment with ononin and JNKi notably decreased the migration and invasion capabilities of MDA-MB-231 cells compared to treatment with JNKi alone. Synergistic administration markedly diminished the expression of ERK1/2, JNK, p38, and their phosphorylated form compared to the administration of JNKi alone. This suggests that the MAPK inhibitor contributed to the down-regulation of MAPK phosphorylation (Fig. 6H and I). These suggests that cotreatment with JNKi and ononin synergistically inhibit malignant phenotypes of MDA-MB-231 cells.

**Fig. 7.**
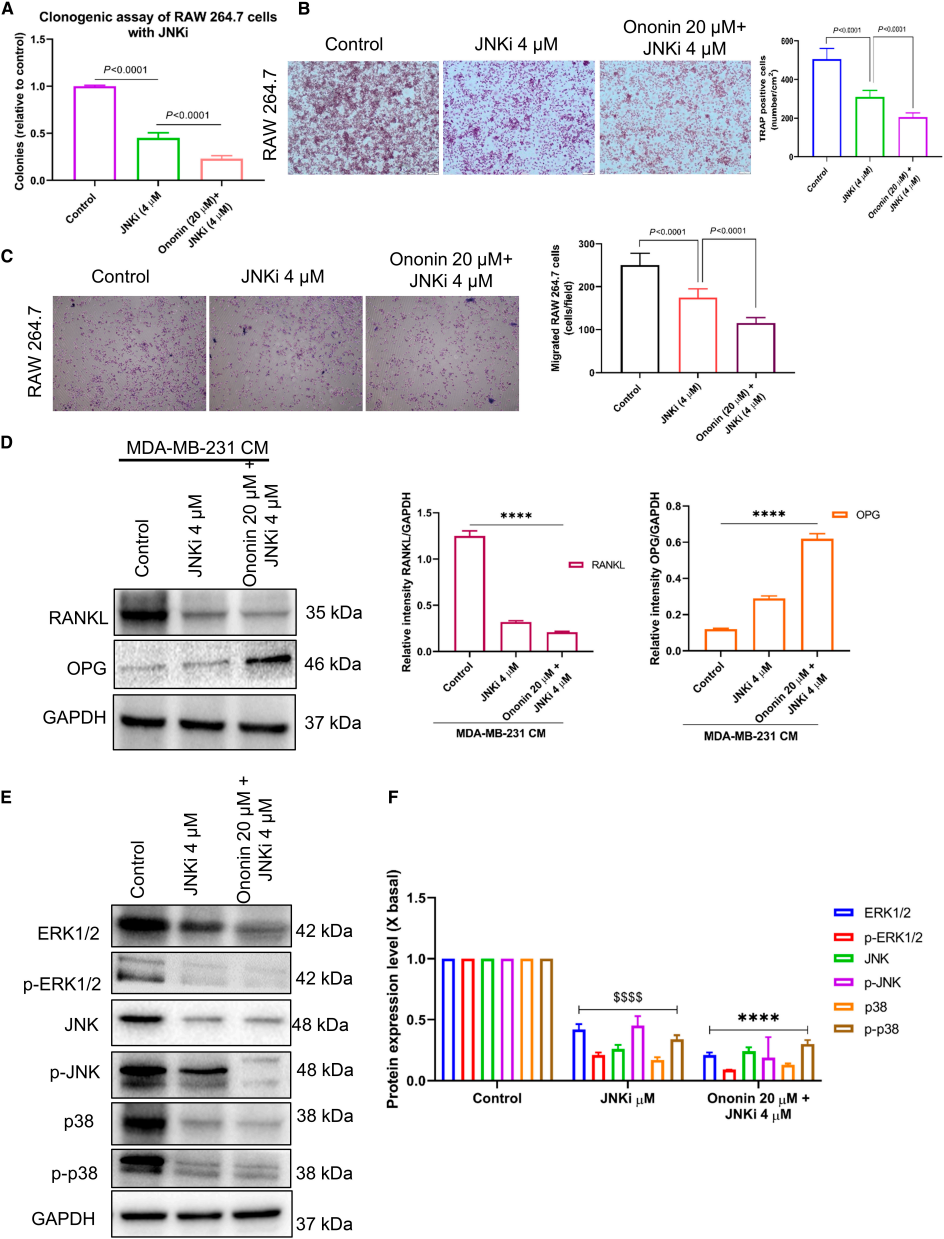
(A) Cotreatment of ononin and JNKi effectively suppresses the formation of colonies in RAW 264.7 cells. (B) Cotreatment of ononin and JNKi inhibits osteoclast differentiation induced by CM, which was analyzed using a TRAP staining assay. (C) Antimigratory effects of the combination of ononin with JNKi were evaluated by Transwell assay. (D) Protein expression levels of RANKL and OPG induced by MDA-MB-231 were investigated. (E and F) The assay of ERK1/2/JNK/p38 pathways was performed using a WB. ^$$$$^ *P* < 0.0001 and ****P < 0.0001.

The concurrent application of ononin and JNKi markedly diminished colony formation (Fig. 7A) and migration capabilities (Fig. 7C) of RAW 264.7 cells activated by CM from MDA-MB-231, compared to JNKi treatment alone or the control group. Additionally, we investigated the impact of ononin and JNKi on osteoclast differentiation utilizing TRAP staining. We noted a substantial decrease of TRAP^+^ cells in the combination of ononin with JNKi compared to JNKi treatment alone (Fig. 7B). Therefore, our results indicate that ononin, particularly in combination with JNKi, may effectively inhibit osteoclast differentiation induced by CM from MDA-MB-231 cells. The results described in Fig. 7D demonstrate that the combination treatment of ononin and JNKi effectively inhibits factors associated with osteolysis, specifically RANKL, while promoting bone formation by increasing levels of OPG. These results suggest that ononin and its synergistic effect with JNKi hold great promise for enhancing bone health by inhibiting osteoclast formation and promoting new bone formation. In addition, synergistic administration markedly down-regulates MAPK pathway phosphorylation compared to the administration of JNKi alone in RAW 264.7 cells treated with CM (Fig. 7E and F). These results suggest that cotreatment with JNKi and ononin has promising potential for inhibiting BM in BC.

### Ononin inhibits PMA-induced cell proliferation, invasion, migration, and osteolytic factors through MAPK pathway

**Fig. 8.**
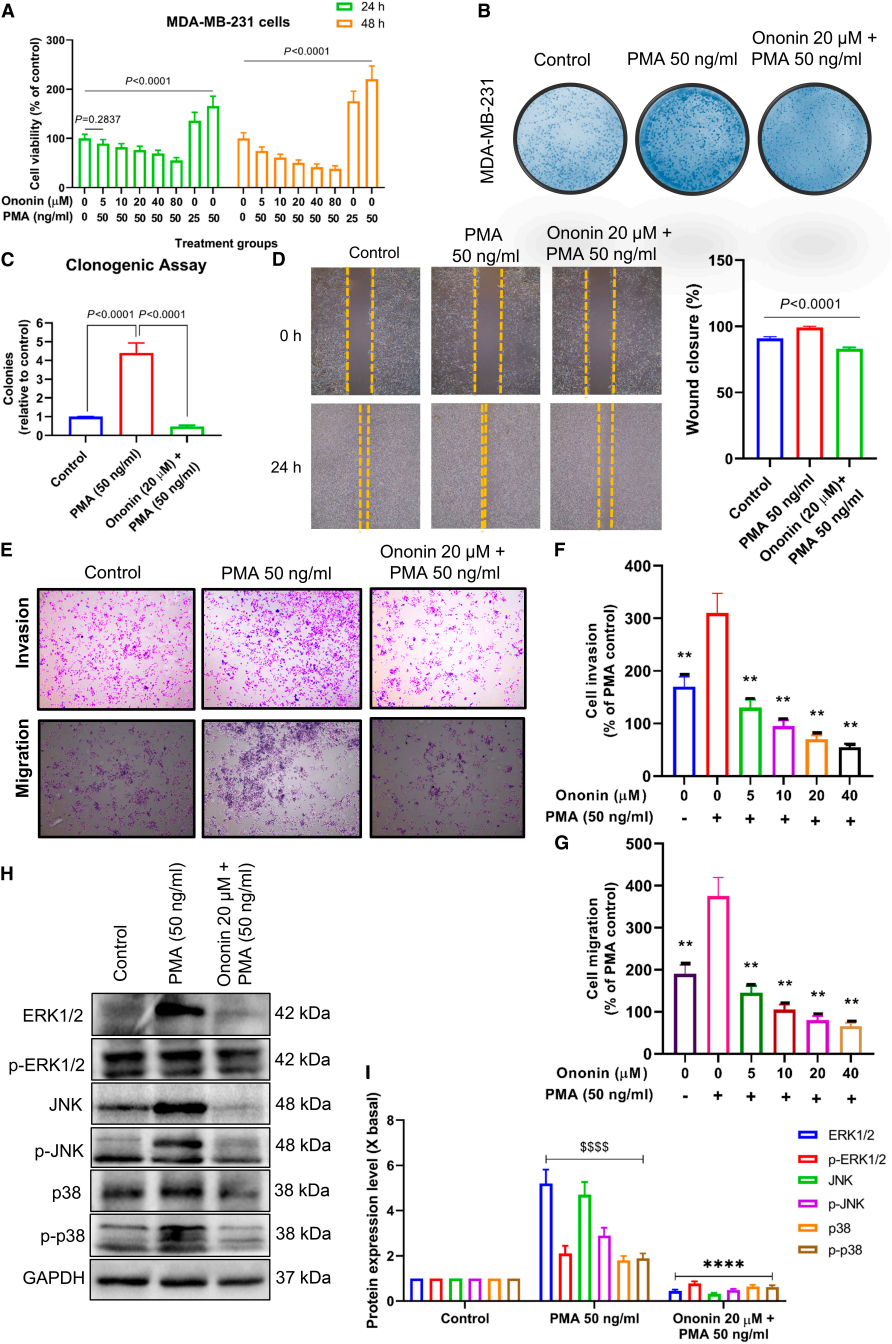
Proliferation, migration, and invasion of MDA-MB-231 cells induced by PMA are inhibited by ononin (A). The MTT assay was conducted in MDA-MB-231 cells. (B and C). Clonogenic assay was performed in MDA-MB-231. (D) The wound-healing assay was utilized to measure the extent of wound closure, quantifying the migratory properties of the cells. (E to G) Non-Matrigel-coated and Matrigel-coated Transwell assay on MDA-MB-231 cells. (H and I) The assay of ERK1/2/JNK/p38 pathways was performed using a WB. ^$$$$^ *P* < 0.0001 and ****P < 0.0001.

Phorbol-12-myristate-13-acetate (PMA) is a ligand that activates the MAPK pathways, promoting cell cycle progression, proliferation, invasion, migration, and metastasis in BC [34,35]. To explore the possibility of ononin-mediated inhibition of PMA-induced TNBC cell metastasis, we exposed MDA-MB-231 cells to PMA (50 ng/ml) with or without ononin for 24 h. Our findings indicated that after 24 h of exposure to PMA, there was a marked enhancement in cell viability, motility, colony formation, and invasive potential. Conversely, ononin administration decreased these PMA-induced effects (Fig. 8A to G).

Building upon an earlier investigation showing that ononin administration can prevent MAPK activation, we conducted further investigations to determine its effects on the phosphorylation of these pathways induced by PMA. WB analysis demonstrated that PMA markedly enhanced the phosphorylation levels of ERK1/2, JNK, and p38 MAPK (Fig. 8H and I). Nonetheless, pretreatment with ononin for 24 h markedly diminished these changes induced by PMA, which indicates that ononin’s suppression of PMA-stimulated metastatic capabilities in TNBC is linked to a down-regulation of MAPK pathways.

**Fig. 9.**
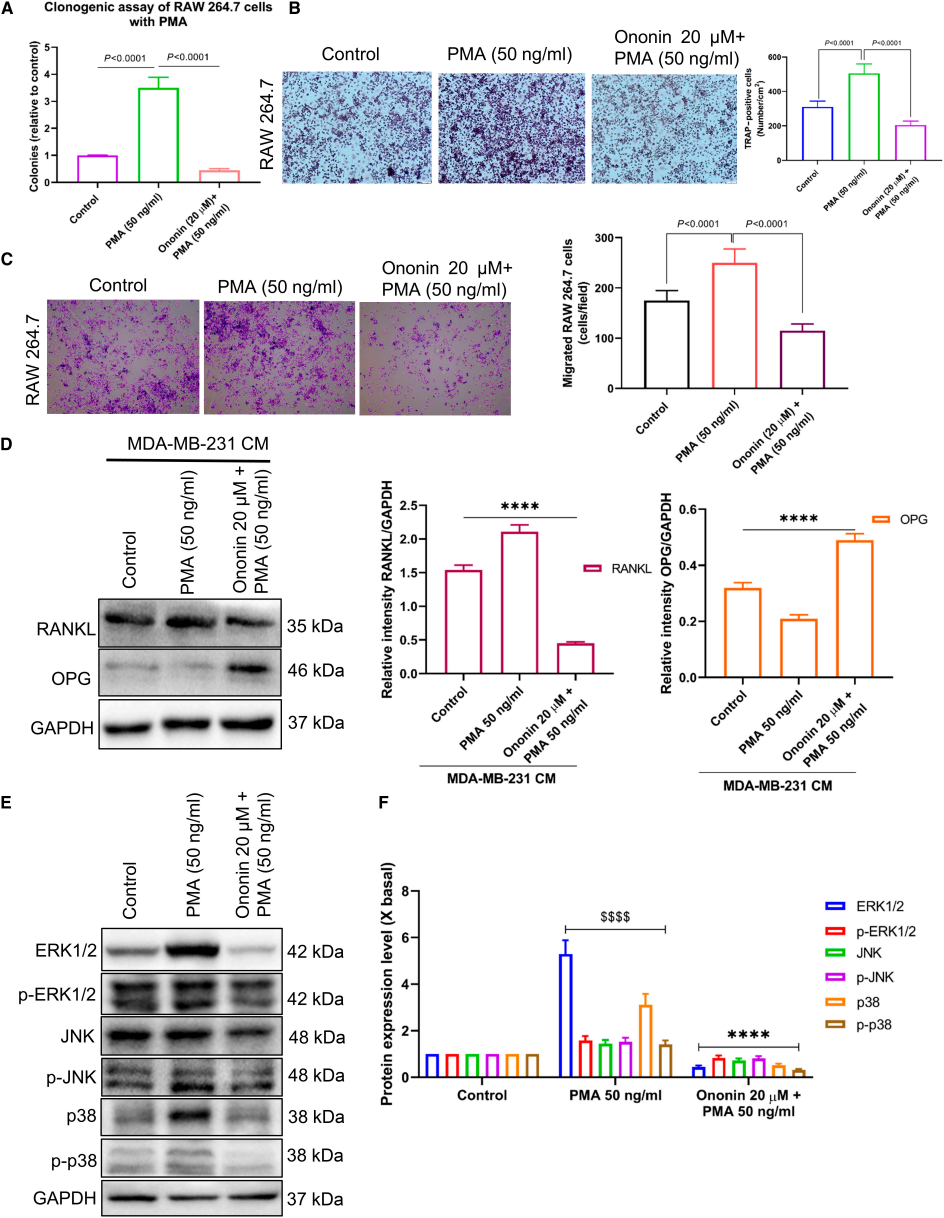
(A) The number of colonies formed under various concentrations of PMA with or without ononin was quantified. (B) Ononin was shown to suppress osteoclast differentiation prompted by PMA in a condition mediated by CM, as determined through TRAP staining analysis. (C) Ononin inhibits PMA-induced migratory effects that were evaluated using RAW 264.7 cells treated with CM. (D) Quantitative analysis of RANKL and OPG protein expression levels. (E and F) The assay of ERK1/2/JNK/p38 pathways was performed using a WB. ^$$$$^ *P* < 0.0001 and ****P < 0.0001.

Next, we further evaluated the impact of ononin on the effects of PMA in RAW 264.7 cells treated with CM. The results, illustrated in Fig. 9A and C, demonstrate that exposure to 50 ng/ml PMA for 24 h led to increased colony formation and migration. Conversely, ononin administration efficiently upturned the clonogenic and migratory effects. Furthermore, ononin markedly reduced the number of TRAP^+^ cells induced by PMA (Fig. 9B). This provided evidence that ononin has the potential to inhibit the differentiation of osteoclasts stimulated by PMA-induced CM.

Figure 9D indicates that administration of ononin, in conjunction with PMA-induced RAW 264.7 cells in CM, effectively inhibits factors associated with osteolysis, specifically RANKL, while promoting bone formation by increasing levels of OPG. These findings suggest that ononin holds great promise for enhancing bone health by inhibiting osteoclast formation and promoting new bone formation. Additionally, WB analysis revealed that ononin treatment inhibits PMA-induced phosphorylation of the MAPK pathway (Fig. 9E and F).

## Discussion

The occurrence of BM in patients with BC poses a noteworthy concern [36,37]. Contemporary therapies targeting the inhibition of osteoclastogenesis have shown effectiveness in reducing skeletal complications and bone damage caused by cancer [4]. Despite the effectiveness of current treatments such as bisphosphonates and denosumab, an important fraction of patients (30 to 50%) continue to develop new BM, experience skeletal complications, and show disease progression [4]. This underscores the critical need for innovative therapeutic strategies in this area [38,39]. This study presents pioneering evidence that ononin suppresses BC-induced osteolytic BM by diminishing the stimulatory impact of BC on osteoclasts and altering the interactions between osteoblasts and osteoclasts.

Evaluating osteoclast differentiation is crucial for comprehending BCBM. The inhibition of osteoclast function is a potential strategy for preventing BM. In this investigation, ononin, an isoflavone glycoside, was examined for its effects and was proved to directly suppress osteoclast differentiation and bone resorption activity. This study represents the inaugural demonstration of ononin’s inhibitory impacts on osteoclast differentiation and bone resorption.

The current study showed that ononin can inhibit osteoclastogenesis and bone degradation mediated by MDA-MB-231 cells. The disruption of normal interactions between osteoclasts and osteoblasts is caused by the presence of metastasized cancer cells in bone [6,40]. Osteoblasts are essential in managing skeletal physiology, functioning both as precursors to osteocytes and as dual regulators in the differentiation of osteoclasts [41]. In our study, MDA-MB-231 cells exhibited elevated RANKL expression coupled with reduced OPG expression, leading to amplified osteoclastogenesis and intensified bone resorption. However, treatment with ononin prevented RANKL up-regulation in MDA-MB-231 cells, leading to a decrease in BC-associated osteoclastogenesis and bone resorption. These results underscore the potential of ononin to correct the disrupted balance between osteoblasts and osteoclasts within the bone microenvironment impacted by BC.

Our research findings indicate that ononin can reduce osteoclast activity and decrease bone loss induced by cancer in mice. Our study demonstrated that control mice had notable bone loss compared to DOX- and ononin-treated tumor-bearing mice, as evidenced by the number of lesions per mouse and the osteolytic area detected using x-ray. Furthermore, histological analysis with TRAP^+^ staining revealed a significant presence of activated osteoclasts in control mice, consistent with observations of bone degradation related to tumor activity. Conversely, groups receiving both DOX and ononin showed increased bone mass, restrained tumor expansion, and reduced numbers of osteoclasts.

Furthermore, our study showed that ononin effectively suppressed BM in BC models, as indicated by a decrease in the fluorescence concentration in the bones and a poorer occurrence of BM. The administration of ononin did not lead to noteworthy weight loss or toxicity in mice, which is consistent with prior studies showing minimal noxiousness of ononin and other polyphenols, even at higher doses [25,42]. Therefore, based on our in vitro and in vivo results, ononin inhibits MDA-MB-231 cell-induced osteolytic lesions by suppressing osteoclast activity.

The MAPK pathway serves as a central signaling hub that regulates a wide range of cellular functions, including cell proliferation, survival, differentiation, and migration. Activation of this pathway plays a crucial role in promoting cancer cell migration and invasion, which are essential processes in the metastatic cascade [43]. By stimulating the expression of genes involved in EMT, MAPK signaling pathways facilitate the acquisition of invasive properties by cancer cells. Clinical studies have consistently shown that abnormal activation of the MAPK pathway is linked to poor prognosis and heightened metastatic potential across various cancer types [44]. The therapeutic potential of targeting components of the MAPK pathway has been underscored by promising results in both preclinical models and clinical trials, highlighting the importance of inhibiting this pathway in the context of metastatic disease [45]. Ongoing research is actively exploring the use of small-molecule inhibitors and monoclonal antibodies that specifically target components of the MAPK pathway as potential antimetastatic agents [46].

The MAPK pathways triggered by RANKL are primary mediators of osteoclastogenesis [47]. RANKL stimulation activates MAPKs associated with osteoclastogenesis [30]. Inhibition of ERK signaling attenuates osteoclast formation [48], whereas the application of dominant-negative JNK obstructs osteoclastogenesis triggered by RANKL [49,50]. Increasing evidence suggests that JNK, a pro-survival oncoprotein, plays a role in tumor progression in several types of cancer, including pancreatic, lung, and BC. The function of JNK in promoting tumor development is influenced by the specific cell context and cell type, as it modulates the signaling pathways involved in tumor initiation, proliferation, and migration [51]. p38 plays a vital role in the initial phases of osteoclastogenesis by regulating the expression of microphthalmia-associated transcription factors [52]. These findings imply that ononin inhibits RANKL/BC cell-induced osteoclast formation via the ERK/JNK/p38 MAPK signaling pathway.

BC cells enhance RANKL signaling by secreting RANKL within the tumor microenvironment [53]. Our research confirms that ononin curtails RANKL- or BC-driven osteoclastogenesis in vitro and obstructs bone deterioration and metastasis in mice with tumors. The inhibitory effects of ononin are likely linked to its suppression of the ERK/JNK/p38 MAPK signaling pathway. These outcomes position ononin as a viable therapeutic candidate for managing cancer-related bone lesions. Additionally, ononin presents benefits over bisphosphonates and denosumab, including cost-effectiveness and fewer adverse effects. This work lays a robust groundwork for further investigations into ononin’s role in combating bone loss associated with BC.

Our study has several limitations. For instance, employing cell lines as study models might not provide a comprehensive representation of the intricate tumor microenvironment in the bone and might not accurately mirror the treatment responses observed in clinical environments. The use of immunodeficient mice in the experiment is another limitation that may restrict the application of our results to immunocompromised model studies. Additionally, the dosage and treatment duration of ononin used in our study may not have been optimal, and the treatment procedures or alternative dosages may have generated dissimilar results. Moreover, the current study focused on the ERK, JNK, and p38 MAPK signaling pathways and did not investigate other possible pathways or mechanisms that may contribute to the experiential effects.

**Fig. 10.**
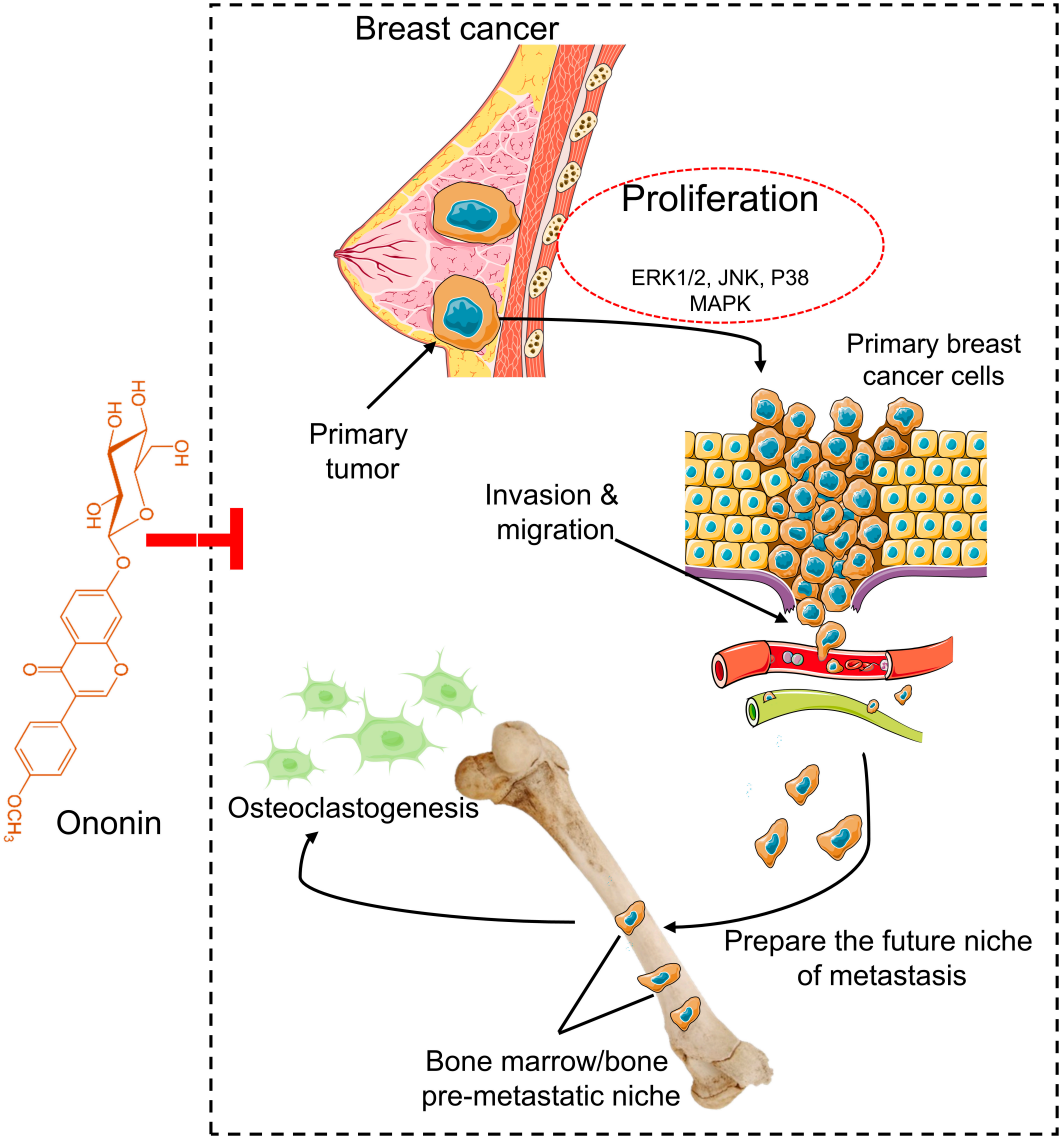
Mechanism of BCBM inhibition by ononin. Ononin targets the ERK1/2/JNK/p38 pathway in TNBC cells, leading to the suppression of cancer cell proliferation, migration, invasion, and ultimately metastasis. In vivo, ononin inhibits TNBC BM by targeting this pathway.

BCBM remains a noteworthy challenge in clinical oncology, and targeted therapies currently have limited effectiveness. Osteoclasts play a crucial role in bone destruction and tumor cell growth in the bones of patients with BCBM, and inhibiting osteoclastogenesis represents a potential approach for preventing or treating BCBM. The current study showed that ononin effectively inhibited the proliferation, colony formation, and metastatic capabilities of MDA-MB-231 cells and suppressed osteoclast formation and osteolysis-associated factors. The in vivo experiments demonstrated that ononin reduced BC cell-induced bone destruction and inhibited the MAPK pathway (Fig. 10). The results of our study indicate that ononin is a harmless and promising agent for BCBM treatment by targeting the MAPK pathway. Further research is necessary to confirm their efficacy and potential in clinical settings. The potential impact of ononin on disease stage classification in BCBM patients should be explored, particularly regarding its influence on metastatic lesion stabilization or reduction. By elucidating how ononin treatment may affect disease progression and response monitoring, clinicians can better assess the therapeutic benefits and tailor treatment strategies for individual patients. In addition, ononin treatment disrupts key processes in the metastatic cascade, such as angiogenesis, immune evasion, and tumor dormancy, elucidating its multifaceted mechanisms.

The emergence of resistance poses a noteworthy challenge in the development of effective therapies for BM. Studies have shown that cancer cells can develop resistance to targeted therapies by modulating key signaling pathways, including MAPK, to promote cell survival and proliferation. Moreover, the activation of compensatory signaling pathways in response to treatment pressure can also contribute to treatment resistance. Understanding these resistance mechanisms is crucial for devising strategies to overcome resistance and enhance the efficacy of ononin treatment. Future research efforts should focus on elucidating the molecular mechanisms underpinning resistance to ononin and exploring combination therapies that can target multiple pathways to mitigate the development of resistance and improve treatment outcomes in BM.

Addressing challenges and opportunities related to the formulation of ononin for clinical use is a key issue. Factors such as bioavailability, stability, and route of administration need to be considered to ensure optimal delivery and efficacy of the drug in treating BCBM [54]. Determining the optimal dose, frequency of administration, and duration of treatment is crucial to achieve therapeutic benefits while minimizing potential side effects [55]. Criteria for selecting patients who are most likely to benefit from ononin therapy must be outlined, considering factors such as tumor characteristics, previous treatment history, and overall health status to personalize treatment strategies and improve patient outcomes. Identifying areas for future investigation, such as pharmacokinetic studies, combination therapies, and biomarker identification, can guide the development of more effective and personalized treatment approaches for BCBM.

### Limitation and future perspectives

1. Conducting additional preclinical studies to assess the long-term efficacy and safety of ononin treatment in various models of BCBM.

2. Exploring potential combination therapies involving ononin and other targeted agents to enhance treatment outcomes and overcome potential resistance mechanisms.

3. Translating the preclinical findings into clinical trials to evaluate the efficacy of ononin in human patients with BCBM, potentially leading to the development of novel treatment strategies for this challenging condition.

4. Investigating the impact of ononin on tumor microenvironment components other than osteoclasts, such as immune cells, to comprehensively understand its effects on BCBM progression.

5. The study primarily focused on the MDA-MB-231 cell line, which may not fully represent BC heterogeneity. Including diverse cell lines could enhance understanding.

## Materials and Methods

### Cell culture

MDA-MB-231 and RAW 264.7 cells were obtained from the American Type Culture Collection (ATCC) (Manassas, VA, USA) at passages 5 to 15. These cells were cultured following the protocol provided by the supplier. The MDA-MB-231 cell line was obtained from a Caucasian woman diagnosed with metastatic BC. This cell line has been classified as a member of the claudin-low molecular subtype.

### Drug treatments

Ononin (catalog no. 111747-200501) was provided by the National Institutes for Food and Drug Control (Beijing, China). The effective concentrations of ononin were established by serially diluting the stock solution in an appropriate medium. Cells were subjected to 50 μM DOX and ononin at concentrations ranging from 5 to 80 μM for various durations ranging from 12 to 144 h, in the cell proliferation assay. For the clonogenic assay, cells were incubated with 5 and 20 μM of ononin for a minimum of 1 week. In the assays of Transwell and wound healing, cells were exposed to a coadministration of ononin and PMA (catalog no. 16561-29-8, Sigma-Aldrich, St. Louis, MO, USA) or CC-401 (JNKi, catalog no. sc-364748, Santa Cruz Biotechnology Inc., TX, USA). BC cells were initially pretreated with ononin for 2 h, and then they were exposed to 50 ng/ml PMA for 24 h. Specifically, in MDA-MB-231 cells, ononin pretreatment lasted 24 h, succeeded by either PMA (50 ng/ml) or JNKi (4 μM) administration for 30 min, to assess the activation states of ERK1/2, JNK, and p38 signaling pathways. To conduct cotreatments with JNKis, BC cells were initially exposed to 4 μM CC-401 (JNKi, catalog no. 395104-30-0, MedChemExpress, NJ, USA) for 30 min. The cells were then treated with 20 μM ononin for 24 h.

### Colony formation, wound-healing, MTT, and Transwell assay

MTT, colony formation, wound-healing, and Transwell assays were conducted according to previously established methods [4,28].

### Western blot

The antibodies and their working concentrations used in the WB experiments are detailed in Table S1.

### Animals

Female BALB/c nude mice (14 to 16 g; 5 to 6 weeks) were sourced from the Laboratory Animal Unit at the University of Hong Kong. A BM assay was performed as previously established [56]. The evaluation of tumor metastasis was conducted using bioluminescence imaging on days 3, 7, 17, and 25. Mice were randomly allocated to 4 groups. The first group, which served as a vehicle control, was administered saline. The second group (positive control) was treated with DOX at a dosage of 0.5 mg/kg orally every 2 d. The final 2 groups were administered ononin at dosages of 10 and 20 mg/kg orally every 2 d, respectively. The Committee on the Use of Live Animals in Teaching and Research approved the animal experiments conducted in this study (CULATR 5286-20).

### Bioluminescence and H&E staining assay

The bioluminescence assay was conducted according to previously established methods [4].

### TRAP staining

Histological sections underwent TRAP staining utilizing the Leukocyte Acid Phosphatase kit (Sigma-Aldrich), and the analysis was carried out with Image-Pro Plus 6.0 software.

### Statistical analysis

Statistical analyses were performed using GraphPad Prism software (version 9.0). Data were expressed as means ± SDs for 3 replicates. One-way analysis of variance (ANOVA) followed by Tukey’s multiple comparison test was used for group comparisons, with statistical significance set at *P* < 0.05.

## Data Availability

The datasets used and/or analyzed in this study are reported in the article, and/or additional files are available from the corresponding authors.

## References

[1] Siegel RL , Giaquinto AN , Jemal A . Cancer statistics, 2024. CA Cancer J Clin. 2024;74(1):12–49. 38230766 10.3322/caac.21820

[2] Ganesan K , Gao F , Zheng C , Xu C , Tang H , Sui Y , Xie C , Chen J . Isoliquiritigenin-infused electrospun nanofiber inhibits breast cancer proliferation and invasion through downregulation of PI3K/Akt/mTOR and MMP2/9 pathways. J Drug Deliv Sci Technol. 2024;96:Article 105609.

[3] Lipton A . Should bisphosphonates be utilized in the adjuvant setting for breast cancer? Breast Cancer Res Treat. 2010;122(3):627–636. 20490653 10.1007/s10549-010-0935-7

[4] Ganesan K , Xu C , Xie C , Sui Y , Zheng C , Gao F , Chen J . Cryoprotective isoliquiritigenin-zein phosphatidylcholine nanoparticles inhibits breast cancer-bone metastasis by targeting JAK-STAT signaling pathways. Chem Biol Interact. 2024;396:Article 111037. 38719172 10.1016/j.cbi.2024.111037

[5] Weilbaecher KN , Guise TA , McCauley LK . Cancer to bone: A fatal attraction. Nat Rev Cancer. 2011;11(6):411–425. 21593787 10.1038/nrc3055 PMC3666847

[6] Sun W , Ge K , Jin Y , Han Y , Zhang H , Zhou G , Yang X , Liu D , Liu H , Liang XJ , et al. Bone-targeted nanoplatform combining zoledronate and photothermal therapy to treat breast cancer bone metastasis. ACS Nano. 2019;13(7):7556–7567. 31259530 10.1021/acsnano.9b00097

[7] Li B , Wang P , Jiao J , Wei H , Xu W , Zhou P . Roles of the RANKL-RANK axis in immunity—Implications for pathogenesis and treatment of bone metastasis. Front Immunol. 2022;13:Article 824117. 35386705 10.3389/fimmu.2022.824117 PMC8977491

[8] Coleman R , Zhou Y , Jandial D , Cadieux B , Chan A . Bone health outcomes from the international, multicenter, randomized, phase 3, placebo-controlled D-CARE study assessing adjuvant denosumab in early breast cancer. Adv Ther. 2021;38(8):4569–4580. 34185259 10.1007/s12325-021-01812-9 PMC8342342

[9] Suva LJ , Washam C , Nicholas RW , Griffin RJ . Bone metastasis: Mechanisms and therapeutic opportunities. Nat Rev Endocrinol. 2011;7(4):208–218. 21200394 10.1038/nrendo.2010.227 PMC3134309

[10] Hodgson K , Orozco-Moreno M , Goode EA , Fisher M , Garnham R , Beatson R , Turner H , Livermore K , Zhou Y , Wilson L , et al. Sialic acid blockade inhibits the metastatic spread of prostate cancer to bone. EBioMedicine. 2024;104:Article 105163. 38772281 10.1016/j.ebiom.2024.105163 PMC11134892

[11] Coleman RE , Seaman JJ . The role of zoledronic acid in cancer: Clinical studies in the treatment and prevention of bone metastases. Semin Oncol. 2001;28(2 Suppl 6):11–16. 10.1016/s0093-7754(01)90260-x 11346860

[12] Onishi T , Hayashi N , Theriault RL , Hortobagyi GN , Ueno NT . Future directions of bone-targeted therapy for metastatic breast cancer. Nat Rev Clin Oncol. 2010;7(11):641–651. 20808302 10.1038/nrclinonc.2010.134

[13] Guise TA . The vicious cycle of bone metastases. J Musculoskelet Neuronal Interact. 2002;2(6):570–572. 15758398

[14] Clezardin P , Coleman R , Puppo M , Ottewell P , Bonnelye E , Paycha F , Confavreux CB , Holen I . Bone metastasis: Mechanisms, therapies, and biomarkers. Physiol Rev. 2021;101(3):797–855. 33356915 10.1152/physrev.00012.2019

[15] Song X , Wei C , Li X . The signaling pathways associated with breast cancer bone metastasis. Front Oncol. 2022;12: Article 855609. 35372035 10.3389/fonc.2022.855609 PMC8965611

[16] Coniglio SJ . Role of tumor-derived chemokines in osteolytic bone metastasis. Front Endocrinol. 2018;9:313. 10.3389/fendo.2018.00313 PMC5999726 29930538

[17] Roodman GD . Mechanisms of disease: Mechanisms of bone metastasis. N Engl J Med. 2004;350(16):1655–1664. 15084698 10.1056/NEJMra030831

[18] Wu X , Li F , Dang L , Liang C , Lu A , Zhang G . RANKL/RANK system-based mechanism for breast cancer bone metastasis and related therapeutic strategies. Front Cell Dev Biol. 2020;8:76. 32117996 10.3389/fcell.2020.00076 PMC7026132

[19] Gong G , Wan Y , Liu Y , Zhang Z , Zheng Y . Ononin triggers ferroptosis-mediated disruption in the triple negative breast cancer both in vitro and in vivo. Int Immunopharmacol. 2024;132:Article 111959. 38554442 10.1016/j.intimp.2024.111959

[20] Gu J , Sun R , Wang Q , Liu F , Tang D , Chang X . Standardized *Astragalus mongholicus* Bunge-*Curcuma aromatica* Salisb. Extract efficiently suppresses colon cancer progression through gut microbiota modification in CT26-bearing mice. Front Pharmacol. 2021;12:Article 714322. 34531745 10.3389/fphar.2021.714322 PMC8438123

[21] Luo LY , Fan MX , Zhao HY , Li MX , Wu X , Gao WY . Pharmacokinetics and bioavailability of the isoflavones formononetin and ononin and their in vitro absorption in Ussing chamber and Caco-2 cell models. J Agric Food Chem. 2018;66(11):2917–2924. 29504397 10.1021/acs.jafc.8b00035

[22] Ye B , Ma J , Li Z , Li Y , Han X . Ononin shows anticancer activity against laryngeal cancer via the inhibition of ERK/JNK/p38 signaling pathway. Front Oncol. 2022;12: Article 939646. 35912256 10.3389/fonc.2022.939646 PMC9334013

[23] Zgórka G , Maciejewska-Turska M , Makuch-Kocka A , Plech T . In vitro evaluation of the antioxidant activity and chemopreventive potential in human breast cancer cell lines of the standardized extract obtained from the aerial parts of zigzag clover (Trifolium medium L.). Pharmaceuticals. 2022;15(6):699. 35745618 10.3390/ph15060699 PMC9229722

[24] Zhou R , Chen H , Chen J , Chen X , Wen Y , Xu L . Extract from Astragalus membranaceus inhibit breast cancer cells proliferation via PI3K/AKT/mTOR signaling pathway. BMC Complement Altern Med. 2018;18(1):83. 29523109 10.1186/s12906-018-2148-2 PMC5845298

[25] Gong G , Ganesan K , Xiong Q , Zheng Y . Antitumor effects of ononin by modulation of apoptosis in non-small-cell lung cancer through inhibiting PI3K/Akt/mTOR pathway. Oxidative Med Cell Longev. 2022;2022:5122448. 10.1155/2022/5122448 PMC9810408 36605098

[26] Cui J , Zheng X , Yang D , Hu Y , An C , Bo Y , Li H , Zhang Y , Niu M , Xue X , et al. Astragali radix total flavonoid synergizes cisplatin to inhibit proliferation and enhances the chemosensitivity of laryngeal squamous cell carcinoma. RSC Adv. 2019;9(42):24471–24482. 35527911 10.1039/c9ra04701h PMC9069756

[27] Sharma U , Sharma B , Mishra A , Sahu A , Mathkor DM , Haque S , Raina D , Ramniwas S , Gupta M , Tuli HS . Ononin: A comprehensive review of anticancer potential of natural isoflavone glycoside. J Biochem Mol Toxicol. 2024;38(6): Article e23735. 38773908 10.1002/jbt.23735

[28] Ganesan K , Xu C , Wu J , du B , Liu Q , Sui Y , Song C , Zhang J , Tang H , Chen J . Ononin inhibits triple-negative breast cancer lung metastasis by targeting the EGFR-mediated PI3K/Akt/mTOR pathway. Sci China Life Sci. 2024;67(9):1849–1866. 38900236 10.1007/s11427-023-2499-2

[29] Song C , Yang X , Lei Y , Zhang Z , Smith W , Yan J , Kong L . Evaluation of efficacy on RANKL induced osteoclast from RAW264.7 cells. J Cell Physiol. 2019;234(7):11969–11975. 30515780 10.1002/jcp.27852

[30] Cheng Y , Liu H , Li J , Ma Y , Song C , Wang Y , Li P , Chen Y , Zhang Z . Monascin abrogates RANKL-mediated osteoclastogenesis in RAW264.7 cells via regulating MAPKs signaling pathways. Front Pharmacol. 2022;13:Article 950122. 35910375 10.3389/fphar.2022.950122 PMC9337785

[31] Kong L , Ma R , Cao Y , Smith W , Liu Y , Yang X , Yan L . Cell cytoskeleton and proliferation study for the RANKL-induced RAW264.7 differentiation. J Cell Mol Med. 2021;25(10): 4649–4657. 33742541 10.1111/jcmm.16390 PMC8107080

[32] Louw-du Toit R , Simons M , Africander D . Progestins and breast cancer hallmarks: The role of the ERK1/2 and JNK pathways in estrogen receptor positive breast cancer cells. J Steroid Biochem Mol Biol. 2023;237:Article 106440. 38048919 10.1016/j.jsbmb.2023.106440

[33] Insua-Rodríguez J , Pein M , Hongu T , Meier J , Descot A , Lowy CM , de Braekeleer E , Sinn HP , Spaich S , Sütterlin M , et al. Stress signaling in breast cancer cells induces matrix components that promote chemoresistant metastasis. EMBO Mol Med. 2018;10(10):Article e9003. 30190333 10.15252/emmm.201809003 PMC6180299

[34] Babu RL , Naveen Kumar M , Patil RH , Kiran Kumar KM , Devaraju KS , Ramesh GT , Sharma SC . Forskolin and phorbol 12-myristate 13-acetate modulates the expression pattern of AP-1 factors and cell cycle regulators in estrogen-responsive MCF-7 cells. Genes Dis. 2019;6(2):159–166. 31194000 10.1016/j.gendis.2018.12.001 PMC6545452

[35] Lacroix M , Haibe-Kains B , Hennuy B , Laes J , Lallemand F , Gonze I , Cardoso F , Piccart M , Leclercq G , Sotiriou C . Gene regulation by phorbol 12-myristate 13-acetate in MCF-7 and MDA-MB-231, two breast cancer cell lines exhibiting highly different phenotypes. Oncol Rep. 2004;12(4):701–707. 15375488 10.3892/or.12.4.701

[36] Liang Y , Zhang H , Song X , Yang Q . Metastatic heterogeneity of breast cancer: Molecular mechanism and potential therapeutic targets. Semin Cancer Biol. 2020;60:14–27. 31421262 10.1016/j.semcancer.2019.08.012

[37] Huang X , Song C , Zhang J , Zhu L , Tang H . Circular RNAs in breast cancer diagnosis, treatment and prognosis. Oncol Res. 2023;32(2):241–249. 38186573 10.32604/or.2023.046582 PMC10765117

[38] Yue Z , Niu X , Yuan Z , Qin Q , Jiang W , He L , Gao J , Ding Y , Liu Y , Xu Z , et al. RSPO2 and RANKL signal through LGR4 to regulate osteoclastic premetastatic niche formation and bone metastasis. J Clin Invest. 2022;132(2):Article e144579. 34847079 10.1172/JCI144579 PMC8759794

[39] Tang Y , Tian W , Zheng S , Zou Y , Xie J , Zhang J , Li X , Sun Y , Lan J , Li N , et al. Dissection of FOXO1-induced LYPLAL1-DT impeding triple-negative breast cancer progression via mediating hnRNPK/β-catenin complex. Research. 2023;6:0289. 38111678 10.34133/research.0289 PMC10726293

[40] Ye F , Dewanjee S , Li Y , Jha NK , Chen ZS , Kumar A , Vishakha , Behl T , Jha SK , Tang H . Advancements in clinical aspects of targeted therapy and immunotherapy in breast cancer. Mol Cancer. 2023;22(1):105. 37415164 10.1186/s12943-023-01805-y PMC10324146

[41] Wang S , Wu W , Lin X , Zhang KM , Wu QL , Luo M , Zhou J . Predictive and prognostic biomarkers of bone metastasis in breast cancer: Current status and future directions. Cell Biosci. 2023;13(1):224. 38041134 10.1186/s13578-023-01171-8 PMC10693103

[42] Zhou T , Zhang A , Kuang G , Gong X , Jiang R , Lin D , Li J , Li H , Zhang X , Wan J , et al. Baicalin inhibits the metastasis of highly aggressive breast cancer cells by reversing epithelial-to-mesenchymal transition by targeting β-catenin signaling. Oncol Rep. 2017;38(6):3599–3607. 29039569 10.3892/or.2017.6011

[43] Morgos DT , Stefani C , Miricescu D , Greabu M , Stanciu S , Nica S , Stanescu-Spinu II , Balan DG , Balcangiu-Stroescu AE , Coculescu EC , et al. Targeting PI3K/AKT/mTOR and MAPK signaling pathways in gastric cancer. Int J Mol Sci. 2024;25(3):1848. 38339127 10.3390/ijms25031848 PMC10856016

[44] Singh SP , Dosch AR , Mehra S , de Castro Silva I , Bianchi A , Garrido VT , Zhou Z , Adams A , Amirian H , Box EW , et al. Tumor cell-intrinsic p38 MAPK signaling promotes IL1α-mediated stromal inflammation and therapeutic resistance in pancreatic cancer. Cancer Res. 2024;84(8):1320–1332. 38285896 10.1158/0008-5472.CAN-23-1200

[45] Brown BA , Myers PJ , Adair SJ , Pitarresi JR , Sah-Teli SK , Campbell LA , Hart WS , Barbeau MC , Leong K , Seyler N , et al. A histone methylation-MAPK signaling axis drives durable epithelial-mesenchymal transition in hypoxic pancreatic cancer. Cancer Res. 2024;84(11):1764–1780. 38471099 10.1158/0008-5472.CAN-22-2945 PMC12032584

[46] Lin W , Szabo C , Liu T , Tao H , Wu X , Wu J . STING trafficking activates MAPK-CREB signaling to trigger regulatory T cell differentiation. Proc Natl Acad Sci USA. 2024;121(29): Article e2320709121. 38985760 10.1073/pnas.2320709121 PMC11260101

[47] Boyle WJ , Simonet WS , Lacey DL . Osteoclast differentiation and activation. Nature. 2003;423(6937):337–342. 12748652 10.1038/nature01658

[48] Pennanen P , Kallionpää RA , Peltonen S , Nissinen L , Kähäri VM , Heervä E , Peltonen J . Signaling pathways in human osteoclasts differentiation: ERK1/2 as a key player. Mol Biol Rep. 2021;48(2):1243–1254. 33486672 10.1007/s11033-020-06128-5 PMC7925492

[49] Ethiraj P , Haque IA , Alford AK , Gou W , Singh T , Sambandam Y , Hathaway-Schrader JD , Reddy SV . Inhibition of NFAM1 suppresses phospho-SAPK/JNK signaling during osteoclast differentiation and bone resorption. J Cell Biochem. 2021;122(10):1534–1543. 34228377 10.1002/jcb.30076 PMC8479865

[50] Wu S , Lu J , Zhu H , Wu F , Mo Y , Xie L , Song C , Liu L , Xie X , Li Y , et al. A novel axis of circKIF4A-miR-637-STAT3 promotes brain metastasis in triple-negative breast cancer. Cancer Lett. 2024;581:Article 216508. 38029538 10.1016/j.canlet.2023.216508

[51] Wu Q , Wu W , Jacevic V , Franca TCC , Wang X , Kuca K . Selective inhibitors for JNK signalling: A potential targeted therapy in cancer. J Enzyme Inhib Med Chem. 2020;35(1): 574–583. 31994958 10.1080/14756366.2020.1720013 PMC7034130

[52] Kim JH , Kim K , Kim I , Seong S , Lee KB , Kim N . BCAP promotes osteoclast differentiation through regulation of the p38-dependent CREB signaling pathway. Bone. 2018;107:188–195. 29223746 10.1016/j.bone.2017.12.005

[53] Tian Y , Gong Z , Zhao R , Zhu Y . Melatonin inhibits RANKL-induced osteoclastogenesis through the miR-882/Rev-erbα axis in Raw264.7 cells. Int J Mol Med. 2021;47(2): 633–642. 33416111 10.3892/ijmm.2020.4820 PMC7797465

[54] Alqahtani MS , Kazi M , Alsenaidy MA , Ahmad MZ . Advances in oral drug delivery. Front Pharmacol. 2021;12: Article 618411. 33679401 10.3389/fphar.2021.618411 PMC7933596

[55] Tomczak S , Kaszuba K , Szkudlarek J , Piwowarczyk L , Jelińska A . Potential use of common administration of emulsion for parenteral nutrition and vinpocetine: Compatibility study and prospect. Meta. 2024;14(8):439. 10.3390/metabo14080439 PMC11356329 39195535

[56] Wang Y , Lei R , Zhuang X , Zhang N , Pan H , Li G , Hu J , Pan X , Tao Q , Fu D , et al. DLC1-dependent parathyroid hormone-like hormone inhibition suppresses breast cancer bone metastasis. J Clin Invest. 2014;124(4):1646–1659. 24590291 10.1172/JCI71812 PMC3973085

