# Supplementary material for: Ononin Inhibits Tumor Bone Metastasis and Osteoclastogenesis By Targeting Mitogen-Activated Protein Kinase Pathway in Breast Cancer
Source: Research (Wash D C). 2024 Dec 16;7:0553. doi: 10.34133/research.0553 (PMC11648741; doi:10.34133/research.0553)
Supplement: Supplementary 1 — Fig. S1 Table S1 [file research.0553.f1.docx]

**Supplementary Materials**

**
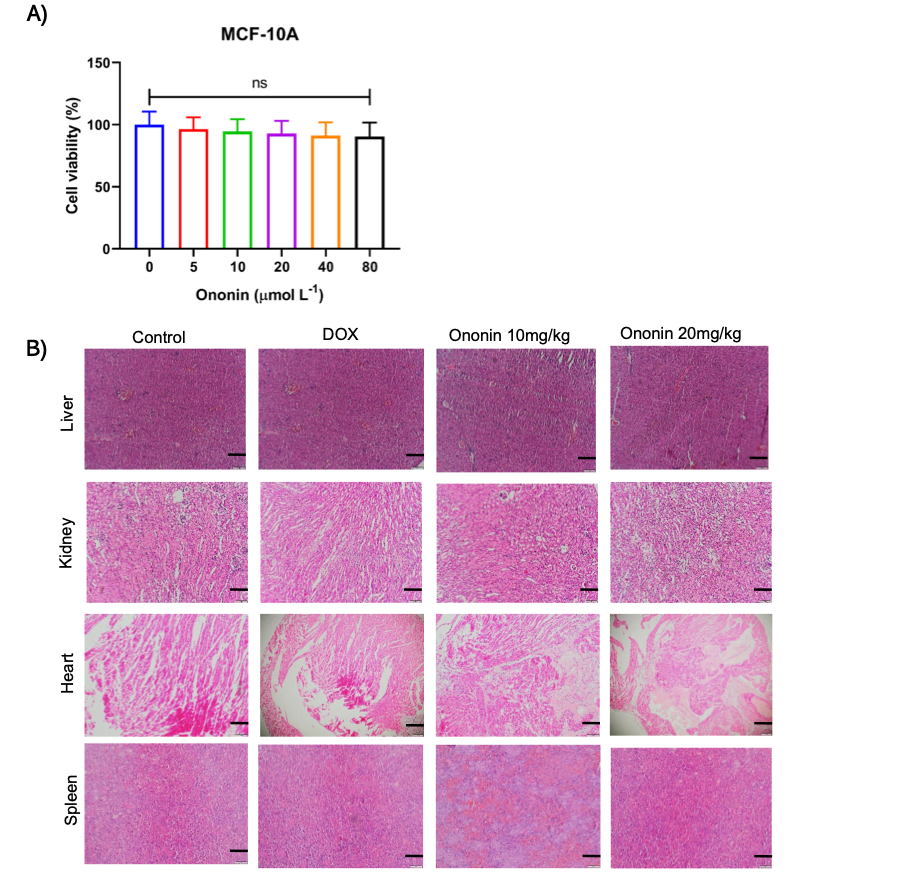
**

**Fig. S1.** Cytotoxic effect of ononin. **(A)** Cytotoxic effect of ononin on normal mammary epithelial cells (MCF-10A). **(B)** Ononin prevents organ toxicity *in vivo*. Representative H&E staining of the liver, kidney, heart, and spleen sections from control and experimental mice (magnification: 4×). No significant differences were observed among the control and treated groups, which indicates that ononin is a safe molecule.

**Table S1.** Antibodies used in the study

| Antibodies | Catalog number | Source | Dilution | Company Name |  |
| --- | --- | --- | --- | --- | --- |
| Primary antibodies | | | | | |
| ERK1/2 | Cat#AP1120 | Rabbit | 1:200 | Abclonal |  |
| p- ERK1/2 | Cat#AP0472 | Rabbit | 1:500 | Abclonal |  |
| JNK | Cat#A4867 | Rabbit | 1:200 | Abclonal |  |
| p-JNK | Cat#AP0631 | Rabbit | 1:200 | Abclonal |  |
| P-38 MAPK | Cat# A16822 | Rabbit | 1:200 | Abclonal |  |
| p-P-38 MAPK | Cat# AP1508 | Rabbit | 1:200 | Abclonal |  |
| N-cad | Cat#A0433 | Rabbit | 1:200 | Abclonal |  |
| MMP-2 | Cat#A6247 | Rabbit | 1:200 | Abclonal |  |
| MMP-9 | Cat#A0289 | Rabbit | 1:200 | Abclonal |  |
| Casp 3 | Cat#A0214 | Rabbit | 1:200 | Abclonal |  |
| Casp 9 | Cat# A11910 | Rabbit | 1:500 | Abclonal |  |
| E-cad | Cat#A3149 | Mouse | 1:200 | Abclonal |  |
| GAPDH | Cat# 2118 s | Rabbit | 1:1000 | Cell Signaling Technology |  |
| RANKL | Cat#A2550 | Rabbit | 1:200 | Abclonal |  |
| OPG | Cat#ab183910 | Rabbit | 1:500 | Abcam |  |
| Secondary antibodies | | | | | |
| Mouse | Cat# 7076 | Horse | 1:500 | Cell Signaling Technology |  |
| Rabbit | Cat# 7074 | Goat | 1:500 | Cell Signaling Technology |  |
